# TANK-Binding Kinase 1 (TBK1) Serves as a Potential Target for Hepatocellular Carcinoma by Enhancing Tumor Immune Infiltration

**DOI:** 10.3389/fimmu.2021.612139

**Published:** 2021-02-18

**Authors:** Yuchuan Jiang, Siliang Chen, Qiang Li, Junjie Liang, Weida Lin, Jinying Li, Zhilong Liu, Mingbo Wen, Mingrong Cao, Jian Hong

**Affiliations:** ^1^ Department of General Surgery, The First Affiliated Hospital, Jinan University, Guangzhou, China; ^2^ Department of Hematology, Peking University Shenzhen Hospital, Peking University, Shenzhen, China; ^3^ Department of Gastroenterology, The First Affiliated Hospital, Jinan University, Guangzhou, China; ^4^ Department of Pathophysiology, School of Medicine, Jinan University, Guangzhou, China

**Keywords:** TANK-binding kinase 1, immune infiltration, inflammation, targeted therapy, hepatocellular carcinoma

## Abstract

**Background:**

Numerous cancer types present the aberrant TANK-binding kinase 1 (TBK1) expression, which plays an important role in driving inflammation and innate immunity. However, the prognostic role of TBK1 and its relationship with immune cell infiltration in hepatocellular carcinoma (HCC) remain unclear.

**Methods:**

The expression and prognostic value of TBK1 was analyzed by Tumor Immune Estimation Resource (TIMER), Kaplan-Meier plotter and Gene Expression Profiling Interactive Analysis (GEPIA), Clinical Proteomic Tumor Analysis Consortium (CPTAC) and further confirmed in the present cohort of patients with HCC. The association between TBK1 and HCC immune infiltrates, and its potential mechanism were investigated *via* analyses of the Tumor Immune Estimation Resource, tumor-immune system interactions database (TISIDB), CIBERSORT, STRING, and Metascape. The effect of TBK1 on immune infiltrates and the therapeutic value of targeting TBK1 were further investigated in a HCC mouse model by treatment with a TBK1 antagonist.

**Results:**

The level of TBK1 expression in HCC was higher than that measured in normal tissues, and associated with poorer overall survival (GEPIA: hazard ratio [HR]=1.80, *P*=0.038; Kaplan–Meier plotter: HR=1.87, *P*<0.001; CPTAC: HR=2.23, *P*=0.007; Our cohort: HR=2.92, *P*=0.002). In addition, high TBK1 expression was found in HCC with advanced TNM stage and identified as an independent poor prognostic factor for overall survival among patients with HCC. In terms of immune infiltration, tumor tissues from HCC patients with high TBK1 expression had a low proportion of CD8^+^ T cells, and TBK1 expression did not show prognostic value in HCC patients with enriched CD8+ T cells. Furthermore, TBK1 expression was positively correlated with the markers of T cell exhaustion and immunosuppressive cells in the HCC microenvironment. Mechanistically, the promotion of HCC immunosuppression by TBK1 was involved in the regulation of inflammatory cytokines. *In vivo* experiments revealed that treatment with a TBK1 antagonist delayed HCC growth by increasing the number of tumor-infiltrating CD8+ T cells.

**Conclusions:**

The up-regulated expression of TBK1 may be useful in predicting poor prognosis of patients with HCC. In addition, TBK1, which promotes the HCC immunosuppressive microenvironment, may be a potential immunotherapeutic target for patients with HCC.

## Introduction

Hepatocellular carcinoma (HCC) is the most common primary liver malignancy and the fourth leading cause of cancer-related death worldwide (1). More than 50% of patients with HCC are diagnosed with advanced disease (2). Immunotherapy represents a promising strategy for many types of advanced cancer (3). The US Food and Drug Administration approved the use of checkpoint inhibitors (nivolumab and pembrolizumab) as a treatment option for advanced HCC (4). However, as a typically inflammation-associated cancer (5), HCC shows a unique immunosuppressive microenvironment enhanced by inflammation-related stromal cells and cytokines (6). This results in lower response and acquired resistance to checkpoint inhibitors (7). Therefore, it is urgent to identify novel therapeutic targets correlated with the HCC immunosuppressive microenvironment.

TANK-binding kinase 1 (TBK1) is a member of the inhibitor of nuclear factor-κB kinase (NF-κB) family (8). Upon receptor-mediated pathogen detection, TBK1 phosphorylation promotes the activation of the NF-κB pathway in the innate immune response (9). An initial study linking TBK1 to cancer found that TBK1 supports oncogenic Ras transformation with coupling innate immune signaling to tumor cell survival (10). Previous studies also demonstrated aberrant TBK1 expression and its pro-tumor effects in multiple cancers, including the promotion of migration and invasion in melanoma (11), AXL-induced epithelial–mesenchymal transition in pancreatic cancer (12), and tamoxifen resistance by increasing the transcriptional activity of estrogen receptor α in breast cancer (13). However, the underlying functions and mechanisms of TBK1 in HCC progression remain uncertain.

Recently, it was reported that TBK1 restrains the activation and migration of T cells, which are the main type of lymphocytes involved in the antitumor immune response (14, 15). Moreover, TBK1 contributed to tumor immunosuppression by down-regulating the expression of co-stimulatory molecules and decreasing T cell-priming activity in dendritic cells (16). However, another study yielded contrary results indicating that TBK1 participated in the activation of stimulator of the interferon genes pathway, enhancing antitumor immunity in the tumor microenvironment (17). Moreover, TBK1 was identified as a promoter of resistance to immunotherapy (9). Of note, inhibition of TBK1 effectively blocked the release of immune-suppressive cytokines and improved the therapeutic efficacy of anti-programmed death-ligand 1 (anti-PD-L1) (18). These findings prompted us to investigate the effects of TBK1 on the immune microenvironment and its potential value in the treatment of HCC.

In the present study, we investigated the correlation of TBK1 expression with prognosis and immune infiltration in patients with HCC. Mechanistically, we constructed TBK1-related gene networks and analyzed their function using bioinformatics tools. Importantly, the roles of TBK1 in HCC progression and immune infiltration were further explored *in vivo* (in immunodeficient and immunocompetent mice) using the TBK1 antagonist GSK8613. Our data revealed that TBK1 predicted poor prognosis in patients with HCC and may be a therapeutic target by attenuating tumor immunosuppression.

## Materials and Methods

### UALCAN and Gene Expression Omnibus (GEO) Database Analysis

UALCAN is a comprehensive and interactive resource for analyzing cancer data (http://ualcan.path.uab.edu/index.html) (19). It provides access to publicly available cancer databases, including The Cancer Genome Atlas (TCGA) and MET500 data set. Moreover, it enables researchers to identify the up- or down-regulated genes in tumors compared with normal tissues, and compare the expression of genes of interest in subgroups, as defined by individual cancer stages, tumor grade, gender, age, nodal metastasis status, TP53 mutation status, and tumor histology. GEO2R is an interactive web tool that enables researchers to analyze the different expression of genes in two or more groups of samples across experimental conditions in a GEO series (20). In the present study, we investigated the levels of TBK1 mRNA expression in different types of cancer and corresponding normal tissues using UALCAN and GEO2R.

### Gene Expression Profiling Interactive Analysis (GEPIA), Kaplan–Meier (KM) Plotter, and Clinical Proteomic Tumor Analysis Consortium (CPTAC) Database Analysis

The online database GEPIA is an interactive web server for the analysis of RNA sequencing expression data from the TCGA and Genotype-Tissue Expression projects, which include 9,736 tumors and 8,587 normal samples (21). The KM plotter is an online available tool for exploring the effect of 54,675 genes on survival in 21 types of cancer. Sources for the databases include the GEO, TCGA, and European Genome-phenome Archive (22). We performed the survival analysis based on TBK1 mRNA expression in 33 different types of cancer using GEPIA and in 21 different types of cancer using the KM plotter. According to the mRNA expression of markers of CD4, CD8 and B cell in HCC tissues, the KM-plotter tool divided the HCC cohort from TCGA into enriched and decreased infiltration of the three types of cell. We used the KM-plotter to investigate the survival time of HCC patients based on the content of CD4, CD8 and B cell (https://kmplot.com/analysis/index.php?p=service&cancer=pancancer_rnaseq). The tool of “auto select best cutoff” (all possible cut off values between the lower and upper quartiles are computed, and the best performing threshold is used as a cutoff) in GEPIA and KM plotter were used to determine the cut-off values in the survival curves (mRNA level). CPTAC is a database established by The National Cancer Institute to promote the understanding of the molecular basis of cancer by applying large-scale proteomic and genomic analyses, or proteogenomics (23). Survival analysis based on TBK1 protein expression in HCC was also performed *via* the CPTAC database. The proteomic data of TBK1 in CPTAC (≤ 0.00368 defined as TBK1 low expression; > 0.00368 defined as TBK1 high expression) were analyzed to select the cut-off value in survival curves (protein level).

### Tumor Immune Estimation Resource (TIMER) Database and Tumor-Immune System Interactions Database (TISIDB) Analysis

TIMER is a comprehensive resource for investigating the interactions between genes of interest and tumor immune interactions in more than 30 types of cancer (https://cistrome.shinyapps.io/timer/) (24). It has incorporated 10,897 samples across 32 types of cancer from TCGA to estimate the abundance of immune infiltrates. The TISIDB is a web portal for the analysis of tumor and immune system interaction; it integrates heterogeneous data types, including literature mining results from the PubMed database, high-throughput screening data, RNA sequencing data of patients with immunotherapy, and TCGA (25). In the present study, we investigated the correlation of TBK1 expression with tumor immune infiltration using TIMER and with tumoral activated CD8^+^ T cells through the TISIDB in the HCC data set. The abundance profile of tumor-infiltrating immune cells in HCC samples from TCGA was calculated using the CIBERSORT computational method (26).

### Gene Ontology (GO) and Kyoto Encyclopedia of Genes and Genomes (KEGG) Pathway Enrichment Analysis

Metascape is an online portal that integrates multiple bioinformatics knowledge bases to provide a comprehensive gene list annotation and analysis resource, especially for functional enrichment, gene annotation, and construction of protein-protein interaction networks (27). Here, we used Metascape to analyze the molecular and functional characteristics of TBK1 and its related genes

### Reagents and Chemicals

TBK1 inhibitor GSK8612 were purchased from Selleck Chemicals (S8872). For *in vitro* experiments, GSK8612 were dissolved in DMSO (Sigma-Aldrich, MO, USA) and further diluted to the required concentration. For *in vivo* experiments, GSK8612 suspension was prepared in 0.5% carboxymethyl cellulose sodium normal saline solution. Antibodies to TBK1 were purchased from Proteintech. Antibodies to α-SMA, CD8α, phospho-TBK1 (p-TBK1) and glyceraldehyde 3-phosphate dehydrogenase (GAPDH) were purchased from Cell Signaling Technology.

### Cell Proliferation and Migration Assay

Hepa1-6 and H22 cell line were gifts from Dr. Limin Zheng (School of Life Sciences, Sun Yat-Sen University, Guangzhou, China). Hepa1-6 cells were cultured in DMEM supplemented with 10% inactivated fetal bovine serum and 1% penicillin-streptomycin (Gibco, USA). Hepa1-6 cells were seeded at 1,000 cells per well in 96-well microplates and incubated in normal growth medium for 24 h. Subsequently, the cells were treated with DMSO or GSK8612 for an additional 24, 48, or 72 h. Cell viability was measured using the Cell Counting Assay Kit-8 (CCK-8; Dojindo, Kumamoto, Japan) according to the manufacturer’s instructions. Cell migration assays were performed on transwell chambers with 8-μm pore-size filters. Cells were trypsinized and resuspended in serum-free medium with DMSO or GSK8612. 250 μl of cell suspension (1 x 10^5^ cells) was added to the upper chambers in a transwell insert, and the upper chambers were then placed into the wells of a 24-well plate. 750 μl culture medium containing 20% fetal bovine serum (FBS) was added to the lower chamber. After transwell inserts were cultured at 5% CO2 at 37°C for 24 h, cells on the top of the membrane were removed with a cotton swabs. Cells attached on the underside of the membrane were fixed and stained with 0.1% crystal violet. After washing with phosphate-buffered saline (PBS), the number of cells was counted in three random microscopic fields under the microscope.

### Histological and Immunohistological Analysis of Liver Sections

Liver and tumor tissues were fixed with 10% formalin, embedded in paraffin and cut into 2 mm sections for staining with hematoxylin-eosin (H&E), Sirius red and immunohistochemistry according to standard procedures (28). For immunohistochemistry (IHC), tumor sections were stained with the appropriate antibodies, and both the intensity and extent of immunostaining were taken into consideration when analyzing the data. The intensity was scored as 0 for negative, 1 for weak staining, 2 for moderate staining and 3 for strong staining. The extent of staining was scored as 0, 0.25, 0.50, 0.75, and 1.00 for less than 5%, 6%–25%, 26%–49%, 50%–74%, and 75%–100% positively stained cells, respectively. The final quantitation of each staining was obtained by multiplying these two values (intensity score × extent score) (29). TBK1 expression was classified as high expression if the score was higher than 1.5; if the score was 1.5 or less, the case was classified as low expression. Two different pathologists who specialize in liver cancer evaluated the results of IHC.

### Western Blotting

The total cellular protein and tissue protein was extracted by RIPA Lysis Buffer (Thermo Fisher Scientific, MA, USA) and RIPA Lysis Buffer (Thermo Fisher Scientific) containing protease inhibitors and phosphatase inhibitors (Thermo Fisher Scientific). The protein concentrations of the cell lysates were measured using a Pierce™ BCA Protein Assay Kit (Thermo Fisher Scientific) and equalized before loading. Equal amount of protein extracts from HCC cells or tissues were separated by SDS–PAGE, and transferred onto polyvinylidene fluoride membranes (Sigma-Aldrich, MO, USA). Immunoblot analyses were carried out using the appropriate antibodies, and the bands were visualized using an SuperSignal™ West Pico PLUS chemiluminescence Substrate (Thermo Fisher Scientific).

### Flow Cytometry

Fresh mouse liver tissues were finely chopped and dissociated into single-cell suspensions. After removal of red blood cells and liver cells, the leukocytes were further purified using a magnetic-activated cell-sorting separator with CD45 magnetic beads (Miltenyi Biotec, CA, USA). After incubation with V450-labeled CD3, PerCP-Cy™-labeled CD4, and V500-labeled CD8 (BD Biosciences, CA, USA), tumor-infiltrated T cells were detected by a flow cytometer (BD LSRFortessa X-20). Gating strategy for CD4^+^ and CD8^+^ T-cell in HCC tissues: lymphocytes were gated by forward and side scatter properties, and then CD4^+^/CD8^+^ T-cells were gated for further analysis (30).

### Enzyme-Linked Immunosorbent Assay (ELISA)

The HCC tissues from mouse model collected above were weighed and homogenized at 4°C. Homogenates were centrifuged at 14,000xg for 10 min at 4°C. Supernatants were transferred to clean microcentrifuge tubes for detection. Specific ELISA kits (Jiangsu Meimian industrial, Jiangsu, China) were used to quantitate IL-6 according to the manufacturer’s instructions.

### *In Vivo* Treatment Studies

Male immunodeficient (BALB/c nude) and immunocompetent (C57BL/6) mice (aged 4–6 weeks) were subjected to carbon tetrachloride (CCl_4_) gavage (40% in 100 μl of olive oil per mouse, volume/volume) for 4 weeks to induce the inflammatory liver microenvironment. Subsequently, mice were injected with 25 μl of HCC cell/Matrigel solution (containing 1×10^6^ Hepa1–6 cells) in the subcapsular region of the liver, and were divided into the control or treatment groups (31). On day 3 following inoculation with tumor cells, the TBK1 antagonist GSK8612 was administered orally at the dose of 5 mg/kg for 7 days. Mice were sacrificed 10 days after HCC implantation. The mice were maintained in the laboratory for animal experimentation in a specific pathogen-free environment with laminar air-flow conditions, a 12-h light-dark cycle, and at a temperature of 22°C–25°C. All animals had free access to standard laboratory mouse food and water. Animal experiments were approved by the Bioethics Committee of Jinan University (China) and performed according to established guidelines.

### Patients and Specimens

Liver samples (n=139) from patients with HCC who underwent hepatectomy were collected in the First Affiliated Hospital of Jinan University. Patient samples were collected and used with the informed written consent of the patient. All liver samples were obtained under protocols approved by the First Affiliated Hospital of Jinan University Office for Protection of Human Subjects.

### Statistical Analysis

The Student’s *t* test was used to compare values between two groups and the ANOVA was employed to compare between subgroups with more than two groups. Overall survival (OS) was calculated by KM survival analysis and log-rank tests. Data were expressed as the mean ± standard deviation of at least three biological replicates. *P* < 0.05 denoted statistical significance. All analyses were performed using the SPSS software (Version 23.0; IBM, Armonk, NY, USA).

## Results

### TBK1 Expression Was Up-Regulated in HCC Tissues

**Figure 1:**
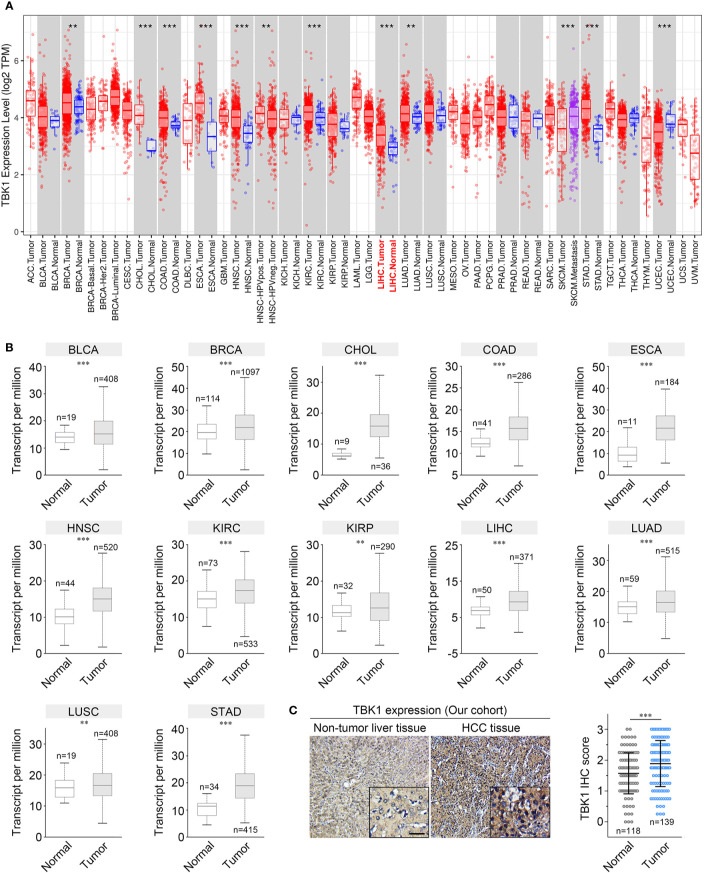
TANK-binding kinase 1 (TBK1) expression levels in human cancer. The levels of TBK1 mRNA expression in different types of human cancer were determined using Tumor Immune Estimation Resource (TIMER) **(A)** and UALCAN **(B)**. **(C)** Representative images of immunohistochemistry (IHC) staining with a TBK1 antibody on HCC tissues (n = 138) and corresponding normal tissues (n = 118) in our cohort. ACC, Adrenocortical carcinoma; BLCA, Bladder urothelial carcinoma; BRCA, Breast invasive carcinoma; BRCA-Basal/Her2/Luminal, Breast invasive carcinoma-Basal/Her2/Luminal; CESC, Cervical squamous cell carcinoma and endocervical adenocarcinoma; CHOL, Cholangiocarcinoma; LIHC, Liver hepatocellular carcinoma; COAD, Colon adenocarcinoma; READ, Rectum adenocarcinoma; DLBC, Lymphoid neoplasm diffuse large B-cell lymphoma; LAML, Acute myeloid leukemia; ESCA, Esophageal carcinoma; GBM, Glioblastoma multiforme; LGG, Brain Lower Grade Glioma; HNSC, Head and neck squamous cell carcinoma; HNSC- HPVneg, Head and neck squamous cell carcinoma-HPVneg; KICH, Kidney chromophobe; KIRC, Kidney renal clear cell carcinoma; KIRP, Kidney renal papillary cell carcinoma; LUAD, Lung adenocarcinoma; LUSC, Lung squamous cell carcinoma; MESO, Mesothelioma; OV, Ovarian serous cystadenocarcinoma; PAAD, Pancreatic adenocarcinoma; PCPG, Pheochromocytoma and Paraganglioma; PRAD, Prostate adenocarcinoma; SARC, Sarcoma; SKCM, Skin cutaneous melanoma; SKCM-Metastasis, Skin cutaneous melanoma- Metastasis; STAD, Stomach adenocarcinoma; TGCT, Testicular Germ Cell Tumors; THCA, Thyroid carcinoma; THYM, Thymoma; UCEC, Uterine Corpus Endometrial Carcinoma; UCS, Uterine Carcinosarcoma; UVM, Uveal Melanoma. *P < 0.05; **P < 0.01; ***P < 0.001.

TIMER and UALCAN were used to analyze the transcriptome-sequencing data from TCGA data set to evaluate the differences in TBK1 expression between tumor and normal samples. The results obtained from TIMER revealed that TBK1 expression was up-regulated in nine types of cancer, including liver hepatocellular carcinoma (LIHC), whereas it was down-regulated in only one type of cancer (**Figure 1A**). Moreover, the results obtained from UALCAN indicated that TBK1 expression was significantly increased in bladder urothelial carcinoma (BLCA), breast invasive carcinoma (BRCA), cholangiocarcinoma (CHOL), colon adenocarcinoma (COAD), esophageal carcinoma (ESCA), head and neck squamous cell carcinoma (HNSC), kidney renal clear cell carcinoma (KIRC), kidney renal papillary cell carcinoma (KIRP), LIHC, lung adenocarcinoma (LUAD), lung squamous cell carcinoma(LUSC), and stomach adenocarcinoma (STAD) (**Figure 1B**).

We further confirmed the expression of TBK1 in multiple human cancers using microarray data sets from GEO. Higher TBK1 expression was found in the subtype of breast cancer, cervical cancer, colorectal cancer, gastric cancer, head and neck cancer, kidney cancer, leukemia, liver cancer, and pancreatic cancer compared with that measured in normal tissues or cells. Meanwhile, TBK1 expression was lower in the subtype of brain cancer (**Table 1**). In addition, the protein level of TBK1 expression in HCC and liver tissues were also determined with immunohistochemistry staining. TBK1 was mainly expressed in hepatocytes and HCC cells, and were also detected in stromal cells. In line with the results obtained from TCGA and GEO databases, the findings of this study indicate that TBK1 expression was significantly increased in HCC tissues (*P*<0.001) (**Figure 1C**).

**Table 1:** Significant changes in TANK-binding kinase 1 (TBK1) expression in cancer versus normal tissue in GEO the database.

Cancer	Subtype	Fold change	*P* value	Adjusted *P* Value	Reference (PMID)	GEO accession number
Breast	Ductal Breast Carcinoma *in situ*	1.434	<0.001	0.009	19187537	GSE14548
Brain	Oligodendroglioma	−1.569	<0.001	<0.001	16616334	GSE4290
Cervical	Cervical Squamous Cell Carcinoma	1.428	<0.001	<0.001	18191186	GSE7410
	Cervical cancer	4.287	<0.001	<0.001	17510386	GSE6791
Colorectal	Rectal carcinoma	1.504	<0.001	<0.001	18171984	GSE8671
Gastric	Gastric mixed adenocarcinoma	1.727	<0.001	<0.001	19081245	GSE13911
Head and neck	Nasopharyngeal carcinoma	1.651	<0.001	<0.001	17119049	GSE12452
Kidney	Clear cell renal cell carcinoma	1.784	<0.001	<0.001	17699851	GSE6344
	Renal pelvis urothelial carcinoma	1.649	<0.001	<0.001	16115910	GSE15641
Leukemia	T-cell prolymphocytic leukemia	2.543	<0.001	0.012	17713554	GSE5788
Liver	Hepatocellular carcinoma	1.512	0.006	0.037	22689435	GSE50579
Pancreatic	Pancreatic ductal adenocarcinoma	1.656	<0.001	<0.001	19260470	GSE15471

The data sets used in the current study has been published in relevant references and can be obtained by GEO accession.

### TBK1 Expression Has Prognostic Significance for Patients With HCC

**Figure 2:**
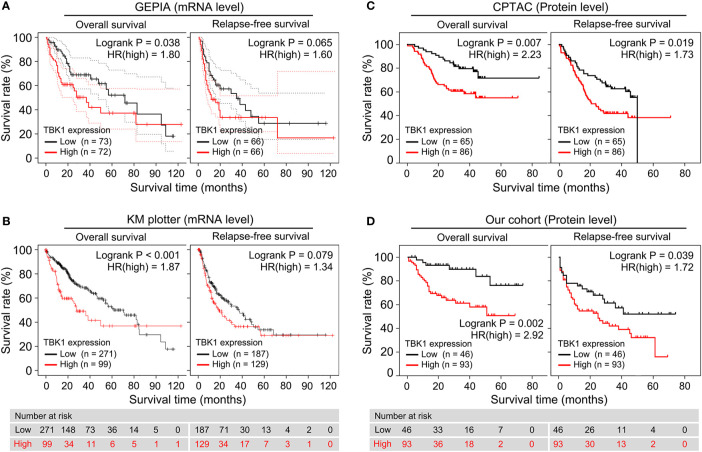
High TANK-binding kinase 1 (TBK1) expression predicted poor prognosis in patients with hepatocellular carcinoma (HCC). **(A, B)** Gene Expression Profiling Interactive Analysis (GEPIA) and the Kaplan–Meier (KM) plotter were used to construct the survival curves of overall survival (OS) and relapse-free survival (RFS) based on the TBK1 mRNA expression in patients with HCC. **(C, D)** The KM survival curves based on TBK1 protein expression in patients with hepatocellular carcinoma (HCC) were determined using Clinical Proteomic Tumor Analysis Consortium (CPTAC) database and our cohort.

We performed a survival analysis based on TBK1 mRNA expression by GEPIA in 33 types of cancer to estimate the influence of TBK1 expression on prognosis in patients with cancer. Although the analysis of relapse-free survival (RFS) in patients with HCC did not reach statistical significance, HCC patients with high TBK1 expression had significantly shorter OS (HR=1.800, *P*=0.038) (**Figure 2A**). In addition, high levels of TBK1 expression were correlated with poorer prognosis of OS in BRCA, ESCA, kidney chromophobe (KICH), KIRP, brain lower grade glioma (LGG), LUAD, Ovarian serous cystadenocarcinoma (OV), pancreatic adenocarcinoma (PAAD), and uveal melanoma (UVM). On the contrary, low levels of TBK1 expression were correlated with poorer prognosis of OS in rectum adenocarcinoma (READ), thymoma (THYM), and uterine carcinosarcoma (UCS) (**Supplementary Figure 1**).

Next, the prognostic potential of TBK1 in different types of cancer was validated by a pan-cancer analysis of 21 types of cancer *via* the KM plotter. Consistent with the results obtained from GEPIA, the KM plotter indicated that high TBK1 expression was correlated with poorer OS (HR=1.870, *P* < 0.001), but not with RFS (**Figure 2B**). Moreover, the findings of the pan-cancer analysis suggested that increased levels of TBK1 expression were associated with worse OS in ESCA, KIRC, LUAD, Pheochromocytoma and Paraganglioma (PCPG), and THYM; however, they were linked to better OS in BLCA, sarcoma (SARC), and thyroid carcinoma (THCA) (**Supplementary Figure 2**).

Furthermore, the association between the levels of TBK1 protein expression and OS or RFS were investigated in the CPTAC database and our cohort. The analysis demonstrated that the protein levels of TBK1 expression were significantly correlated with poorer OS (CPTAC: HR=2.23, *P* = 0.007; Our cohort: HR=2.92, *P*=0.002) and RFS (CPTAC: HR=1.73, *P*=0.019; Our cohort: HR=1.72, *P*=0.039) in patients with HCC (**Figures 2C, D**).

### TBK1 Expression Correlated With Clinicopathological Characteristics and Was Identified as the Independent Prognostic Factor for OS Among Patients With HCC

**Figure 3:**
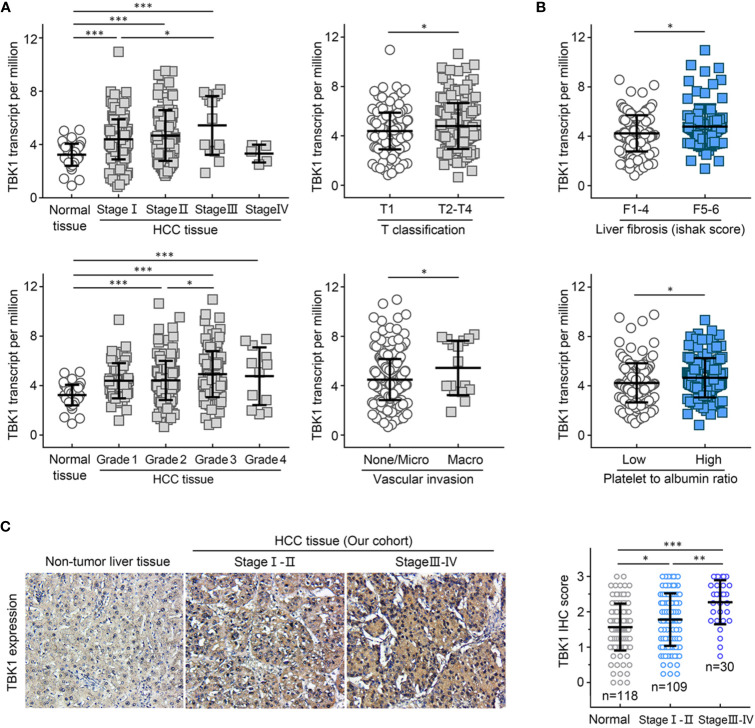
TANK-binding kinase 1 (TBK1) expression was associated with clinicopathological characteristics of patients with hepatocellular carcinoma (HCC). The HCC data set from The Cancer Genome Atlas (TCGA) was used to analyze the levels of TBK1 expression based on the clinical parameters of HCC (TNM stage, grade, T classification, and vascular invasion) **(A)**, and inflammation indicators of patients with HCC (liver fibrosis and platelet-to-albumin ratio) **(B)**. **(C)** The expression levels of TBK1 in different stages of patients with HCC from the present cohort (n=139). *P < 0.05; **P < 0.01; ***P < 0.001.

We analyzed the TBK1 expression based on eight widely recognized clinicopathological parameters of the HCC data set from TCGA, including age, gender, alpha-fetoprotein (AFP), tumor stage, tumor grade, T classification, vascular invasion, liver fibrosis, and the value of platelet-to-albumin ratio. Compared with normal liver tissues, TBK1 expression was markedly increased in HCC classified as Stages I–IV or Grades 1–4. In addition, higher TBK1 expression was found in Stage III HCC versus Stage I and Grade 3 HCC versus Grade 2 (**Figure 3A**). Moreover, patients with a more advanced T classification (*P* = 0.020), severer vascular invasion (*P* = 0.031), higher degree of liver fibrosis (*P* = 0.017), and higher value of platelet-to-albumin ratio (*P* = 0.027) tended to have higher mRNA expression levels of TBK1 (**Figures 3A, B**). Meanwhile, there was no significant association between TBK1 expression and age, sex, or AFP value in patients with HCC (data not shown). We further examined the correlation of levels of TBK1 protein expression in HCC patients with the mentioned clinicopathological characteristics in the present cohort. The analysis demonstrated that increased TBK1 expression was associated with higher degree of platelet-to-albumin ratio, liver fibrosis and tumor stage (**Supplementary Table 1**, **Figure 3C**). These data suggested that HCCs with higher TBK1 expression were more aggressive.

Furthermore, the HCC data set from TCGA and the present cohort were used to determine the independent prognostic potential of TBK1 expression for OS by univariate and multivariate Cox regression analyses. In the HCC data set from TCGA, the univariate analysis indicated that vascular invasion (HR = 1.982, *P* = 0.029), advanced stage (HR = 2.066, *P* = 0.022), and high TBK1 expression (HR = 2.784, *P* = 0.002) significantly contribute to the poor OS. Importantly, the multivariate analysis demonstrated that high expression of TBK1 was an independent risk factor for poor OS in patients with HCC (HR = 2.473, *P* = 0.009) (**Table 2**). In addition, the analysis of present cohort by Cox regression consistently showed the independent prognostic potential of TBK1 expression for OS in patients with HCC (**Supplementary Table 2**). The above results indicated that high levels of TBK1 expression led to poor prognosis and may promote tumor progression in patients with HCC.

**Table 2:** Univariate and multivariate Cox regression analyses of TANK-binding kinase 1 (TBK1) mRNA expression for overall survival (OS) in patients with hepatocellular carcinoma (HCC) from The Cancer Genome Atlas (TCGA) data set.

Characteristics	OS (n=169)			
	Univariate analysis		Multivariate analysis	
	Hazard	*P* value	1Hazard	*P* value
**Age (year)**				
≥60 *vs.* <60	1.696 (0.926–3.106)	0.087		
**Gender**				
Male *vs.* female	0.606 (0.335–1.098)	0.099		
**Platelet to albumin ratio**				
High *vs.* Low	1.264 (0.688–2.322)	0.450		
**Liver fibrosis**				
Cirrhosis vs non-cirrhosis	1.117 (0.603–2.031)	0.744		
**AFP**				
≥400 *vs.* <400	1.319 (0.677–2.571)	0.415		
**Vascular invasion**				
Yes *vs.* No	1.982 (1.074–3.658)	**0.029**	1.544 (0.818–2.917)	0.180
**Tumor grade**				
3+4 *vs.* 1 + 2	1.584 (0.882–2.846)	0.123		
**Tumor stage**				
III+IV *vs.* I+II	2.066 (1.110–3.846)	**0.022**	1.923 (1.027–3.601)	**0.041**
**TBK1 expression**				
High *vs.* Low	2.784 (1.438–5.395)	**0.002**	2.473 (1.253–4.881)	**0.009**

The parameter including age, gender, platelet-to-albumin ratio, liver fibrosis, alpha-fetoprotein (AFP), vascular invasion, tumor grade, tumor stage, and TBK1 expression in HCC were used for univariate Cox regression analyses and significant parameters were included in further multivariate Cox regression analyses. Bold values denote statistical significance at the p < 0.05 level.

### Poor Prognosis of HCC Patients With High TBK1 Expression Was Attributed to the Decreased Levels of Tumor-Infiltrating CD8^+^ T Cells

**Figure 4:**
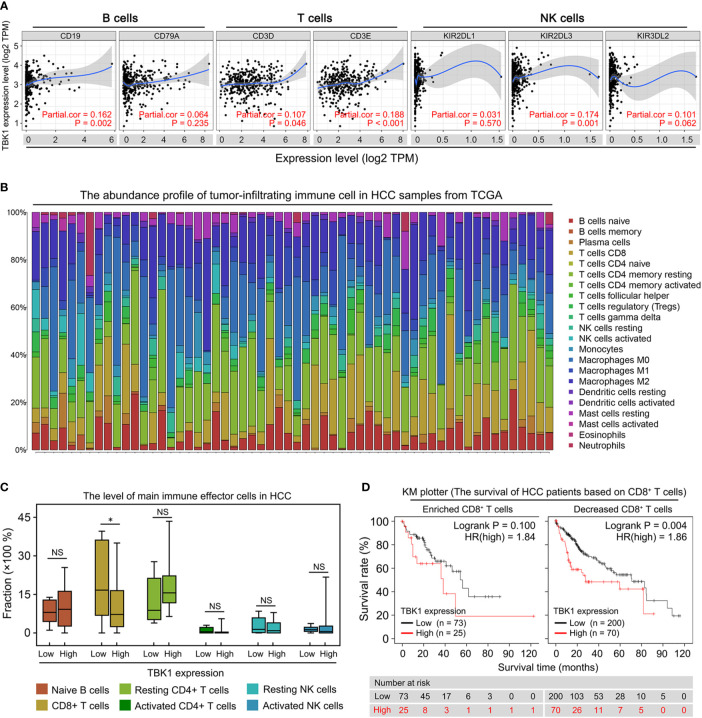
Correlation of TANK-binding kinase 1 (TBK1) expression with tumor immune infiltration in patients with hepatocellular carcinoma (HCC). **(A)** Tumor Immune Estimation Resource (TIMER) was used to analyze the correlation of TBK1 expression with the markers of immune effector cells [B, T, and natural killer (NK) cells]. **(B)** 22 tumor-infiltrating immune cells in HCC samples were estimated using the CIBERSORT algorithm. **(C)** The proportion of main immune effector cells in HCC tissues with high and low TBK1 expression. **(D)** Kaplan–Meier overall survival (OS) curve of high and low TBK1 expression in HCC based on the number of tumor-infiltrating CD8^+^ T cells. NS, not significant; *P < 0.05.

Liver fibrosis and the platelet-to-albumin ratio (**Figure 3B**) are important indicators of liver inflammation, which results in impaired antitumor immune response (5, 32). Therefore, the association between TBK1 expression and degree of immune infiltration in HCC was further investigated in this study. We analyzed the correlation between TBK1 expression and immune marker genes (33) of B, T, and natural killer (NK) cells, which have been identified as important immune effector cells exerting the antitumor response in HCC (14, 34). The data indicated that TBK1 expression was significantly correlated with two markers of T cells (CD3D and CD3E), one marker of B cells (CD19), and one marker of NK cells (KIR2DL3) (**Figure 4A**). Moreover, we further investigated the correlation between TBK1 expression and immune markers of different functional T cells including CD4^+^ T cells, CD8^+^ T cells, Th1 cells, Th2 cells, Tfh cells, and Th17 cells. The results revealed that the TBK1 expression level was significantly correlated with most immune marker sets of T cell in HCC (**Supplementary Figure 3A**). The landscape of tumor-infiltrating immune cells was obtained using the CIBERSORT algorithm, and 22 types of immune cell profiles in patients from the HCC data set of TCGA were constructed to further confirm the association of TBK1 expression with the immune effector cells in this disease (**Figure 4B**). The analysis demonstrated that patients with high TBK1 expression had significantly higher proportions of CD8^+^ T cells. However, there were no significant differences detected in the infiltration levels of B, CD4^+^ T, and NK cells (**Figure 4C**).

We performed a prognosis analysis of TBK1 expression in the immune cells subgroup *via* the KM plotter to examine whether the poor prognosis of HCC patients with high TBK1 expression is related to immune infiltration. The results showed that TBK1 overexpression in HCC samples with enriched or decreased B cells, and enriched or decreased CD4^+^ T cells was a significant indicator of poor prognosis (**Supplementary Figures 3B, C**). However, high TBK1 expression predicted poor prognosis in patients with decreased CD8^+^ T cells, but not in those with enriched CD8^+^ T cells (**Figure 4D**). The above data suggested that high TBK1 expression in HCC contributed to tumor progression and poor prognosis at least partly owing to the decreased number of CD8^+^ T cells.

### TBK1 Expression Is Significantly Correlated With the HCC Immunosuppressive Microenvironment

**Figure 5:**
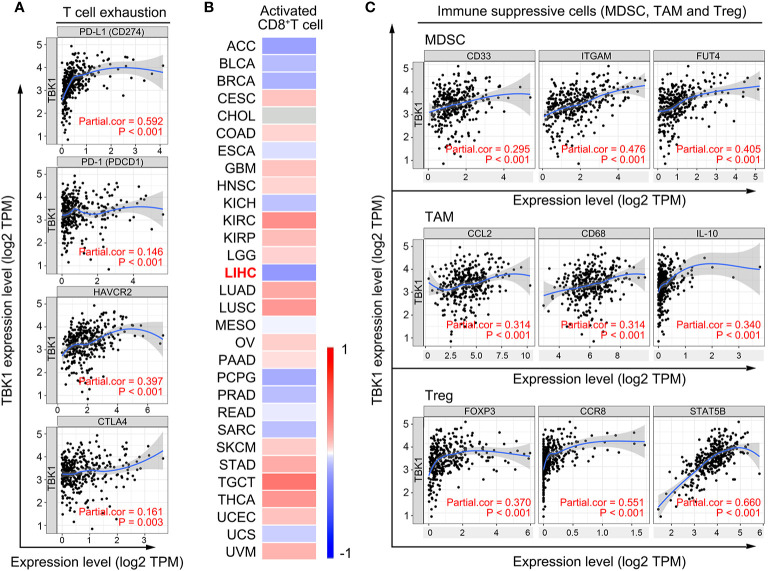
TANK-binding kinase 1 (TBK1) expression is significantly correlated with the markers of immunosuppressive molecules and cells. **(A)** Correlation of TBK1 expression with immunosuppressive molecules (PD-L1, PD-1, HAVCR2, and CTLA4) involved in T cell exhaustion. **(B)** Correlation of TBK1 expression with activated CD8^+^ T cells in different types of cancer. **(C)** Correlation of TBK1 expression with markers of immunosuppressive cells (MDSC, TAM, and Treg).

The decreased number and impaired function of CD8^+^ T cells are mostly resulted by the immunosuppressive molecules and cells in tumor microenvironment (35, 36). Therefore, we used TIMER to investigate the correlation of TBK1 expression with immunosuppressive molecules, the immune checkpoints, involved in T cell exhaustion (37). The analysis suggested that the level of TBK1 expression was positively correlated with the PD-L1 (r = 0.592, *P* < 0.001), hepatitis A virus cellular receptor 2 (HAVCR2; r=0.397, *P* < 0.001), programmed cell death protein 1 (PD-1; r = 0.146, *P* = 0.006), and cytotoxic T lymphocyte-associated antigen-4 (CTLA4; r = 0.161, *P* = 0.003) (**Figure 5A**). The expression of these immune checkpoints is rapidly up-regulated upon T cell activation, and contributes to the deterioration of T cell function (36). Subsequently, we analyzed the correlation of TBK1 expression with the activation of CD8^+^ T cells by TISIDB, and found that the activated CD8^+^ T cell was negatively correlated with TBK1 expression in LIHC data set (r = - 0.211, *P* < 0.001) (**Figure 5B**). Moreover, myeloid-derived suppressor cell (MDSC), tumor-associated macrophage (TAM) and regulatory T cell (Treg) are the main immunosuppressive cells in HCC microenvironment (38). The data from TIMER demonstrated the immune marker sets (37, 39) of MDSC (CD33, ITGAM, FUT4), TAM (CCL2, CD68, IL-10) and Treg (FOXP3, CCR8, STAT5B) were significantly correlated with the TBK1 expression (**Figure 5C**).

### TBK1 Is Involved in the Functional Network of Inflammatory Cytokines

TBK1-related genes with similar expression patterns were examined using the STRING (functional protein association networks) to better understand the underlying mechanisms of the effects of TBK1 expression on immune infiltration. According to the results, we incorporated the up-regulated top 40 proteins-encoding genes that mostly correlated with TBK1 expression for further analysis. The 40 protein-encoding genes are shown below: RELA, IRF3, TAX1BP1, RNF135, TRAF3, OPTN, UBC, IFI16, IKBKE, TRAF2, SQSTM1, LY96, TICAM2, SIKE1, TICAM1,IRF7, IKBKG, NLRP4, TANK, NLRC3, DDX3X, ZBP1, TRAF5, IFIH1, AZI2, DDX58, PRKDC, DTX4, DDX41, CALCOCO2, TRIM25, TNFAIP3, PTPN11, TMEM173, TLR3, EXOC2, TLR4, MAVS, STAT6, and TRAF6 (**Supplementary Figure 4**).

**Figure 6:**
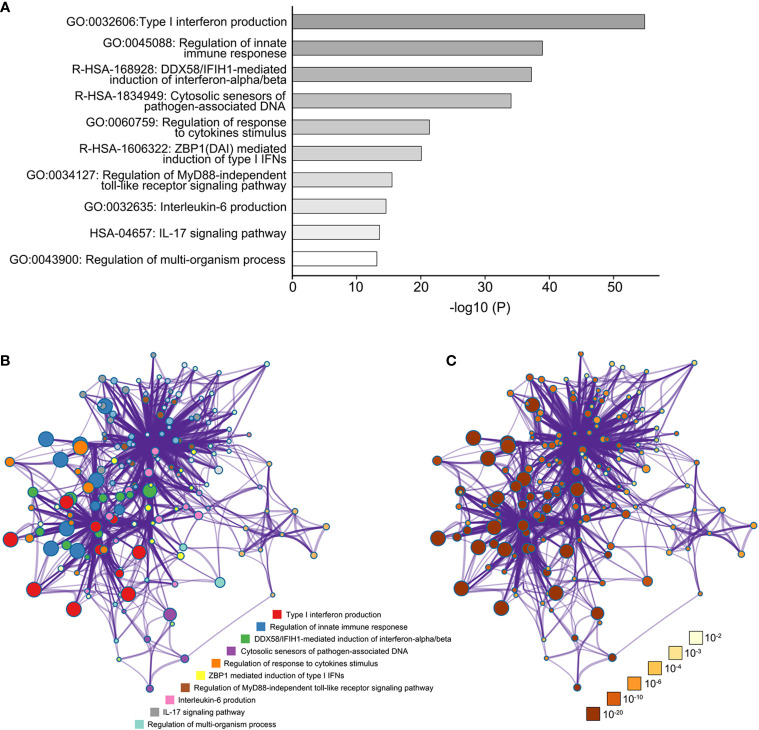
The function network of TANK-binding kinase 1 (TBK1) and TBK1-related genes. **(A)** The gene ontology (GO) and Kyoto Encyclopedia of Genes and Genomes (KEGG) enriched terms colored according to *P*-values. **(B)** Network of GO and KEGG enriched terms colored according to clusters. **(C)** Network of GO and KEGG enriched terms colored according to *P*-values.

Subsequently, the biological functions and pathway enrichment of TBK1, and its related genes were predicted and explored by GO and KEGG approaches using Metascape (**Figure 6A**). Network of GO and KEGG enriched terms colored according >to clusters and P-values were also shown (**Figures 6B, C**). The results suggested that the majority of biological functions and pathways were involved in the inflammatory response of anti-infection (GO:0045088: Regulation of innate immune response; R-HSA-168928: DDX58/IFIH1-mediated induction of interferon-alpha/beta; R-HSA-1834949: Cytosolic sensors of pathogen-associated DNA; GO:0034127: Regulation of MyD88-independent toll-like receptor signaling pathway). These results were consistent with the property of TBK1 gene. More importantly, the production and regulated pathway of inflammatory cytokines were enriched in the function network of TBK1 and its related genes (GO:0032606: Type I interferon production; GO:0032635: IL-6 production; HSA-04657: IL-17 signaling pathway). Type I interferon, IL-6, and IL-17 promote the up-regulation of immunosuppressive molecular and accumulation of immunosuppressive cells in cancer (40–42). Therefore, the results suggested that TBK1-regulated inflammatory cytokines may promote the immunosuppressive microenvironment of HCC, as a clear example of inflammation-related cancer.

### TBK1 Antagonist Attenuates HCC Progression by Enhancing Tumor Immune Infiltration

A previous study reported that TBK1 resulted in tumor immunosuppression and may be therapeutically beneficial to patients, in an effort to augment tumoral T-cell infiltration. However, more investigations on the role of TBK1 in immune-competent animals with tumor are warranted (8). Therefore, we further assessed whether TBK1 promotes HCC progression by decreasing immune infiltration, and investigated the potential immunotherapeutic value of targeting TBK1 by treatment with a TBK1 antagonist.

**Figure 7:**
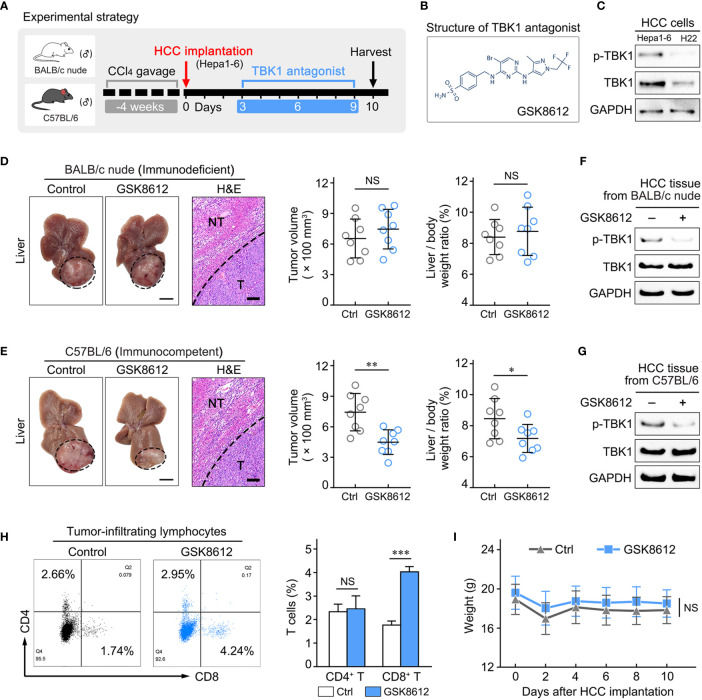
Treatment with a TANK-binding kinase 1 (TBK1) antagonist delayed hepatocellular carcinoma (HCC) growth by increasing the number of tumor-infiltrating CD8+ T cells. **(A)** Experimental design to investigate the effect of the TBK1 antagonist on tumor progression in the orthotopic HCC mouse models with chronic liver inflammation. **(B)** Expression of TBK1 and p-TBK1 in the mouse-derived HCC cell lines (H22 and Hepa1–6). **(C)** Structure of the TBK1 antagonist GSK8612. **(D, E)** Statistical analysis of tumor volume and the liver/body weight ratio, as well as representative images of tumor morphology and hematoxylin-eosin (H&E) staining of liver tissue *in vivo* at the endpoint. **(F, G)** The effect of GSK8612 on TBK1 activation in HCC tissues was detected by western blotting. **(H)** Levels of tumor-infiltrating CD4^+^ and CD8^+^ T cells in HCC tissues obtained from immunocompetent mice. **(I)** Weight changes in immunocompetent mice treated with or without GSK8612. Thin scale bars, 5 mm. Bold scale bars, 200 μm. NT, Non-tumor liver tissue; T, Tumor; NS. not significant; *P < 0.05; **P < 0.01; ***P < 0.001.

We detected the level of TBK1 activation (Phosphorylated TBK1, p-TBK1) in human HCC tissues and non-tumor liver tissues by western blotting, indicating that p-TBK1 was significantly up-regulated in HCCs compared with non-tumor liver tissues (**Supplementary Figure 5A**). Most cases of human HCC arise in fibrotic or cirrhotic livers which is characterized chronic unresolved inflammation. Thus, an orthotopic HCC model that recapitulates the pathological features of human HCC (**Supplementary Figure 5B**) were established using BALB/c nude (immunodeficient) and C57BL/6 mice (immunocompetent) with chronic liver inflammation (**Figure 7A**). The HCC mouse models treated with GSK8612, a novel and highly selective TBK1 antagonist, were sacrificed and liver tissues were harvested for further analysis (**Figures 7A, B**). Two strains of mouse-derived HCC cell lines were tested for TBK1 and p-TBK1 expression, and Hepa1–6 cells with higher level of TBK1 activation were used in the current study (**Figures 7A, C**). We found that the GSK8612 did not have an effect on HCC growth in BALB/c nude mice, whereas it significantly attenuated HCC growth in C57BL/6 mice (**Figures 7D, E**). Western blotting demonstrated a decreased TBK1 activation in HCC tissues of immunodeficient and immunocompetent mice after treatment with GSK8612 (**Figures 7F, G**). The degrees of infiltration of CD4^+^ and CD8^+^ T cells in the tumors of immunocompetent mice were examined and indicated that the number of tumor-infiltrating CD8^+^ T cells was markedly increased after treatment with the TBK1 antagonist (**Figure 7H**). In addition, the TBK1 antagonist resulted in the decreased level of α-SMA^+^ myofibroblasts in non-tumor liver tissues and IL-6 in tumor tissues demonstrated by IHC staining and ELISA (**Supplementary Figure 5C, D**). However, the difference of CD8^+^ T cells in non-tumor liver tissues with or without therapy was not observed (**Supplementary Figure 5C**). The increased level of tumor-infiltrating CD8^+^ T cells after treatment with GSK8612 were also confirmed by IHC staining (**Supplementary Figure 5C**). Besides, there were no significant differences in body weight observed between the two groups (**Figure 7I**). Meanwhile, we investigated the effects of GSK8612 on Hepa1-6 proliferation and migration *in vitro*. The results of CCK8 and Transwell assay showed that the growth rate and migratability of Hepa1-6 were not significantly affected by GSK8612 (**Supplementary Figures 5E, F**). These data suggested that TBK1 contributes to HCC progression by promoting immunosuppression and is a potential therapeutic target in patients with HCC.

## Discussion

As an atypical inhibitor of the NF-κB protein kinase, TBK1 mediates the inner immune response induced by signals from pattern-recognition receptors (PRRs) detecting pathogen-associated molecular patterns (9). Besides, TBK1 possesses important functions in the regulation of immune tolerance and adaptive immune responses. Recent studies investigating the function of TBK1 have expanded their focus on cancers, demonstrating the promoting effect of TBK1 on tumor immunosuppression and therapeutic potential of targeting TBK1 (18, 43). In the present study, we reported that variations in the levels of TBK1 expression were associated with prognosis in different types of cancer. In addition, high TBK1 expression was found in more aggressive tumors and identified as an independent poor prognostic factor for OS among patients with HCC. More importantly, TBK1 expression was positively correlated with a decreased number of tumor-infiltrating CD8+ T cells and increased immunosuppressive markers in patients with HCC. Treatment with the TBK1 antagonist attenuated the HCC progression *in vivo* by enhancing the infiltration of CD8+ T cells in the tumor. Thus, the present study demonstrated the prognostic value of TBK1 expression and immunotherapeutic potential of targeting TBK1 in patients with HCC.

The critical role of TBK1 in tumorigenesis and aberrant TBK1 expression in cancer were reported in previous studies (43–45). In this study, data from TCGA and GEO databases consistently demonstrated the up-regulated levels of TBK1 expression in BRCA, HNSC, KIRC, LIHC, and STAD compared with those measured in normal tissues. Furthermore, GEPIA and the KM plotter indicated the significant value of TBK1 expression as a prognostic biomarker in 17 types of cancer. Differentially expressed genes are involved in count for the molecular mechanisms of biological conditions (46). Therefore, the up-regulated TBK1 expression, which is predictive of poor prognosis, may contribute to tumor progression especially in BRCA, KIRC, and LIHC. Consistent with our results, other studies reported that ectopic TBK1 expression accelerated the growth of BRCA by phosphorylating estrogen receptor α (13), and hyperactivated TBK1 was essential for maintaining p62 stability and the oncogenic phenotype of KIRC (47). However, there is limited knowledge regarding the effect of TBK1 on HCC progression. We further investigated the association with clinicopathological parameters and prognostic potential of TBK1 expression to provide more insight into the pathologic role of TBK1 in HCC progression. The results indicated higher expression of TBK1 in patients with more advanced TNM stage, and identified high TBK1 expression as an independent risk factor for poor OS in patients with HCC. These findings suggest that TBK1 could be used as the prognostic biomarker for patients with HCC, and may play an important role in HCC progression.

HCC occurs mostly in a background of chronically inflamed liver, which enhances the induction of antigen-specific tolerance and suppression of immune response to HCC (48). Owing to the correlation of TBK1 expression with inflammation indicators (liver fibrosis, platelet-to-albumin ratio), the present study investigated the effects of TBK1 expression on HCC immune infiltration. The lower number of tumor-infiltrating CD8^+^ T cells results in impaired host immune defense against HCC progression and poor prognosis (49, 50). Our data further revealed a decreased number of CD8^+^ T cells in HCC with high TBK1 expression, and no significant prognostic value of TBK1 expression in HCC patients with enriched tumoral CD8+ T cells. Thus, it is reasonable to hypothesize that high TBK1 expression leads to HCC progression and poor prognosis by reducing the infiltration of CD8^+^ T cells. In addition, recent studies revealed the effect of TBK1 on promoting immunosuppression in lung and cervical cancer (43, 51). This study also observed that TBK1 expression was significantly correlated with the marker genes of the HCC immunosuppressive microenvironment (**Figure 5**), and its potential mechanism was involved in inflammatory cytokines (type I interferon, IL-6, and IL-17). It has been reported that IL-6 promotes the polarization of monocytes recruited by tumor cells into TAM (52) and the amplification of MDSCs in tumor microenvironment (42), IL-17 enhanced the expression of PD-1 and HAVCR2 in tumor-infiltrated CD8^+^ T cells (53). Although Type I interferon exerts a direct inhibitory effect on tumor growth, it is able to induce immunosuppression through Treg, MDSC accumulation, and PD-L1 up-regulation in a manner of sustained stimulation (54, 55). Collectively, these data suggest that TBK1 induces HCC immunosuppression by sustaining the inflammatory phenotype and promotes HCC progression.

Owing to variable effects on the immune microenvironment in the state of chronic liver inflammation (48), it is important to explore the role of TBK1 in HCC immune infiltration *in vivo*. This study utilized a TBK1 antagonist to treat the orthotopic HCC model established using BALB/c nude and C57BL/6 mice with chronic liver inflammation. The results indicated that treatment did not delay HCC growth in BALB/c nude mice, which is characterized by defective immune responses especially for the T cell-mediated response (56). However, treatment significantly attenuated HCC progression in immunocompetent C57BL/6 mice, accompanied by increased tumoral CD8^+^ T cell infiltration. These data confirmed the role of TBK1 in HCC promotion by decreasing immune infiltration. The HCC cell promotes the inflammatory environment *via* releasing inflammatory cytokines and recruiting the tumor-associated macrophages which amplified the inflammatory response (57–59). In addition, HCC-derived cancer-associated fibroblast contributes to the production of PD-L1^+^ neutrophils by IL-6, impairing the T-cell function and fostering immunosuppression (60). We now report the decreased level of IL-6 in HCC tissues treated by TBK1 antagonist (**Supplementary Figure 5D**) as well as the TBK1 expression in HCC and tumor stroma (**Supplementary Figures 5G, H**). These data suggested that TBK1 antagonist may modulate the immunosuppressive microenvironment by inhibiting the secretion of inflammatory cytokines in HCC cells and cancer-associated fibroblasts. Furthermore, consistent with the other study (61), our results showed the suppression of the activation of hepatic stellate cells and liver fibrosis by TBK1 antagonist (**Supplementary Figure 5C**). Due to the promoting effect of hepatic fibroinflammatory condition on the tumor immunosuppression (5), it is possible that the TBK1 antagonist attenuated the HCC immunosuppression by reducing the fibrosis and inflammatory environment of liver. Previous studies demonstrated the potential value of TBK1 as an immunotherapeutic target for the treatment of cancer (16, 18). Nevertheless, the application of small molecules targeting TBK1 was restricted by its selectivity (8). The recently developed GSK8612, a novel potent and highly selective TBK1 antagonist (62), was used in this study and presented an inhibitory effect on HCC. In addition, the absence of significant weight loss indicative of adverse drug reactions (63) in treated mice partially demonstrated the safety of GSK8612 (**Figure 7I**). These results propose that targeting TBK1 by GSK8612 has potential value as immunotherapy for HCC. Recent reports showed that anti-PD-1/anti-PD-L1-based combination therapy represented a promising strategy for HCC (64), and targeting TBK1 boosted the efficacy of anti-PD-1/anti-PD-L1 in various types of cancer (16, 18). Hence, further studies are warranted to investigate the efficacy of immunotherapy, combining the targeting of TBK1 with administration of immune checkpoint inhibitors, for HCC.

In summary, we demonstrated that increased expression of TBK1 may be useful in predicting the poor prognosis of patients with HCC. Moreover, this study revealed the effect and mechanism of TBK1 on promoting HCC by decreasing immune infiltration, and potential value of targeting TBK1 as an immunotherapy strategy for HCC.

## Data Availability Statement

The original contributions presented in the study are included in the article/supplementary material. Further inquiries can be directed to the corresponding author.

## Ethics Statement

The animal study was reviewed and approved by Bioethics Committee of Jinan University (China). Written informed consent was obtained from the individual(s) for the publication of any potentially identifiable images or data included in this article.

## Author Contributions

YJ, MC, and JH conceived and designed the study. YJ, SC, QL, and JiL performed the experiments. WL, JuL, and ZL collected the clinical samples of each patient. YJ and JH analyzed the data and designed the figure. YJ and JH drafted the manuscript. MW and MC revised the manuscript. All authors contributed to the article and approved the submitted version.

## Funding

This study was funded by the Flagship specialty construction project-General surgery (711003), National Natural Science Foundation of China (81672320 and 81871987), Fundamental Research Funds for the Central Universities (21620106), Science and Technology Program of Guangzhou, China (201704020128), and Guangzhou Science and Technology Program (202002030087).

## Conflict of Interest

The authors declare that the research was conducted in the absence of any commercial or financial relationships that could be construed as a potential conflict of interest.

## Supplementary Material

Supplementary Figure 1 GEPIA indicated the prognostic significance of TBK1 expression for OS in 12 types of cancer. **(A–L)** Kaplan–Meier curves of OS based on TBK1 expression in diverse types of cancer.

Supplementary Figure 2 The KM plotter indicated the prognostic significance of TBK1 expression for OS in 8 types of cancer. **(A–H)** KM curves of OS comparing the high and low expression of TBK1 in different types of cancer.

Supplementary Figure 3 The correlation of TBK1 expression with the marker of T cells and prognostic potential of TBK1 expression in patients with HCC based on immune infiltration. **(A)** TIMER was used to analyze the correlation of TBK1 expression with the markers of CD4^+^ and CD8^+^ T cells as well as Th1, Th2, Tfh and Th17 cells. Comparison of Kaplan–Meier OS curves of high and low TBK1 expression in HCC based on tumor-infiltrating B cells **(B)** or tumor-infiltrating CD4^+^ T cells **(C)**.

Supplementary Figure 4 Co-expressed genes with TBK1 among patients with HCC.

Supplementary Figure 5 TBK1 antagonist improved the immune infiltrates in HCC and attenuated liver fibrosis and tumor inflammation. **(A)** The expression of TBK1 and p-TBK1 in human HCC tissues and non-tumor liver tissues. **(B)** The pathological features of liver tissue from HCC mouse model. **(C)** Representative images of IHC staining with α-SMA and CD8 in liver tissues from control and treatment group (Left panel); statistical analysis of their IHC score (Right panel). **(D)** The level of IL-6 in HCC tissues were examined by ELISA. **(E)** The CCK8 and **(F)** Transwell assays used to measure the effect of GSK8612 on Hepa1-6 proliferation and migration. **(G)** TBK1 expression in tumor stroma of human HCC tissues. **(H)** In the liver tissues of C57BL/6 mouse model, TBK1 expression in tumor stroma indicated by Sirius red staining. NT = Non-tumor liver tissue, T = Tumor, NS = not significant; *, *P* < 0.05; **, *P* < 0.01; ***, *P* < 0.001.
