# Supplementary figures and images for: TANK-Binding Kinase 1 (TBK1) Serves as a Potential Target for Hepatocellular Carcinoma by Enhancing Tumor Immune Infiltration
Source: Front Immunol. 2021 Feb 18;12:612139. doi: 10.3389/fimmu.2021.612139 (PMC7930497; doi:10.3389/fimmu.2021.612139)

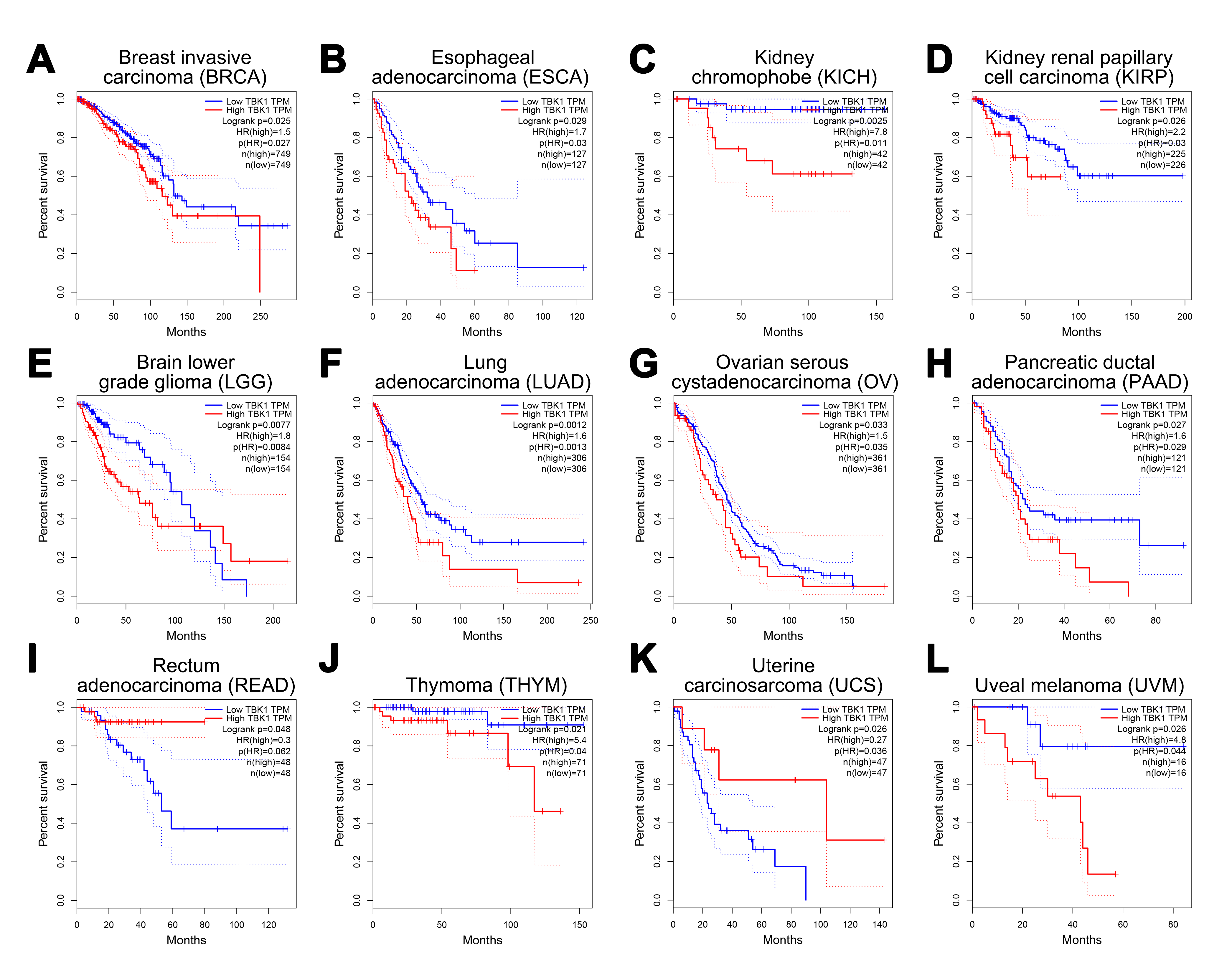

Supplement: Supplementary Figure 1 — GEPIA indicated the prognostic significance of TBK1 expression for OS in 12 types of cancer. (A–L) Kaplan–Meier curves of OS based on TBK1 expression in diverse types of cancer. [file Image_1.tif]

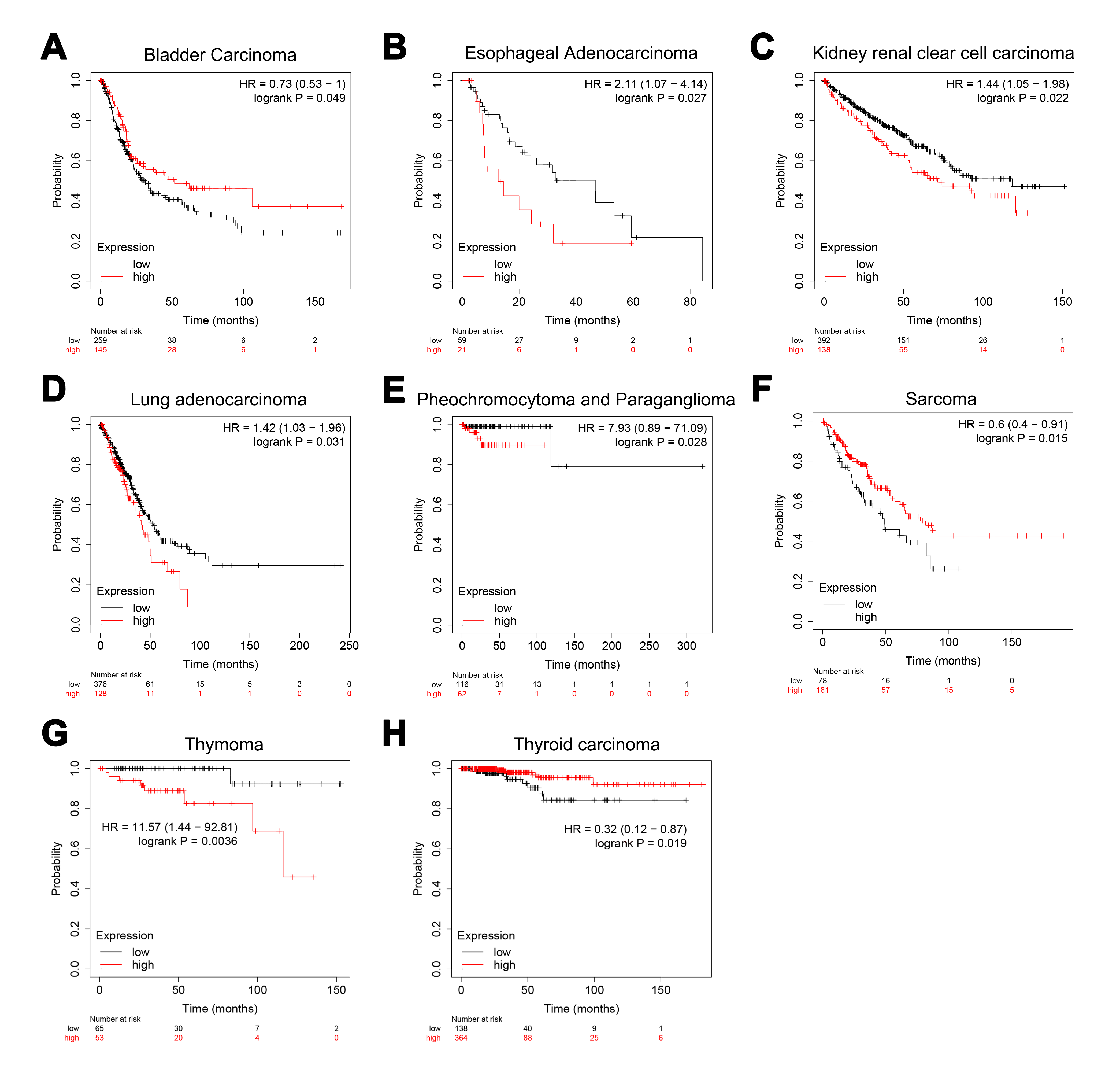

Supplement: Supplementary Figure 2 — The KM plotter indicated the prognostic significance of TBK1 expression for OS in 8 types of cancer. (A–H) KM curves of OS comparing the high and low expression of TBK1 in different types of cancer. [file Image_2.tif]

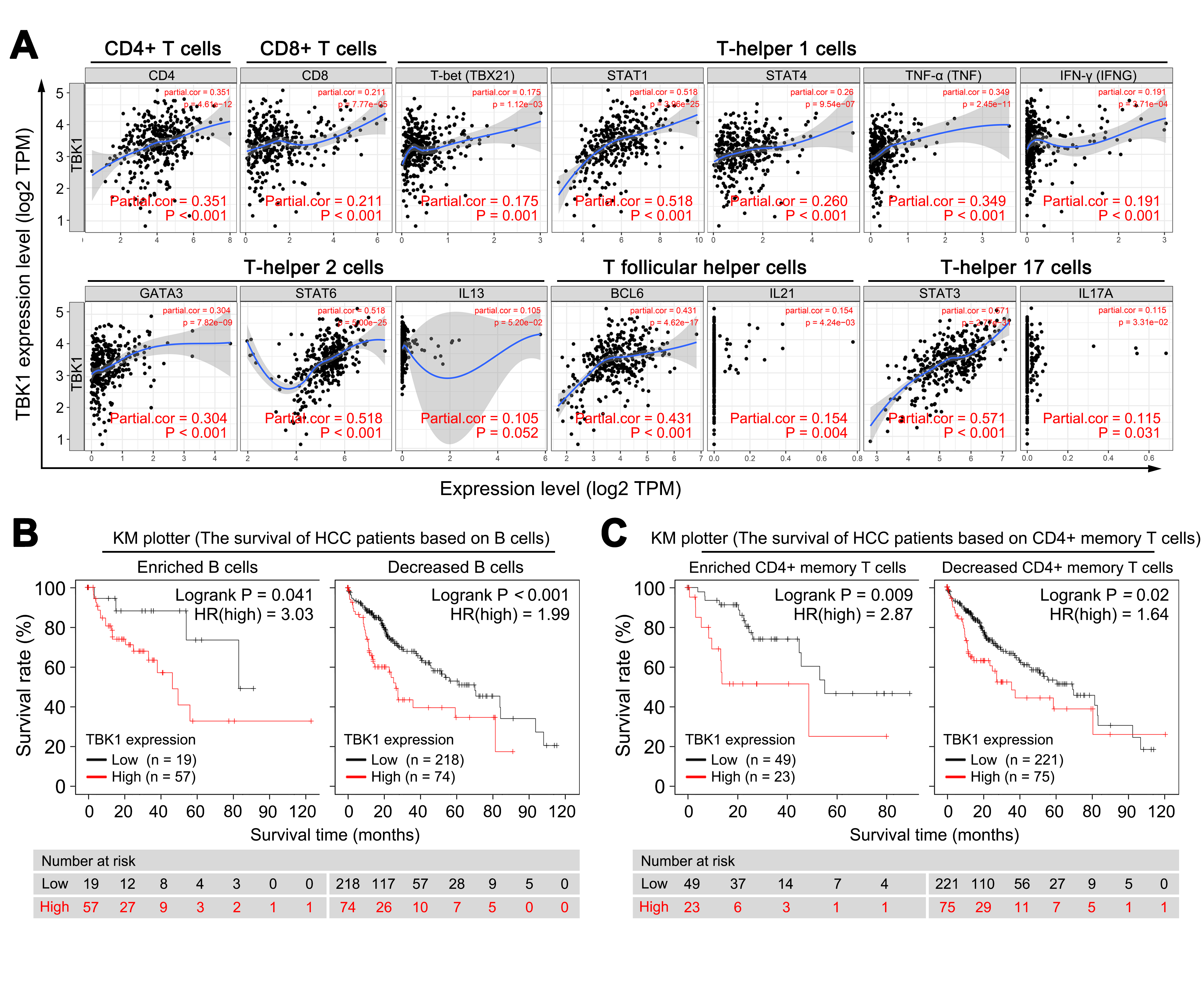

Supplement: Supplementary Figure 3 — The correlation of TBK1 expression with the marker of T cells and prognostic potential of TBK1 expression in patients with HCC based on immune infiltration. (A) TIMER was used to analyze the correlation of TBK1 expression with the markers of CD4+ and CD8+ T cells as well as Th1, Th2, Tfh and Th17 cells. Comparison of Kaplan–Meier OS curves of high and low TBK1 expression in HCC based on tumor-infiltrating B cells (B) or tumor-infiltrating CD4+ T cells (C). [file Image_3.tif]

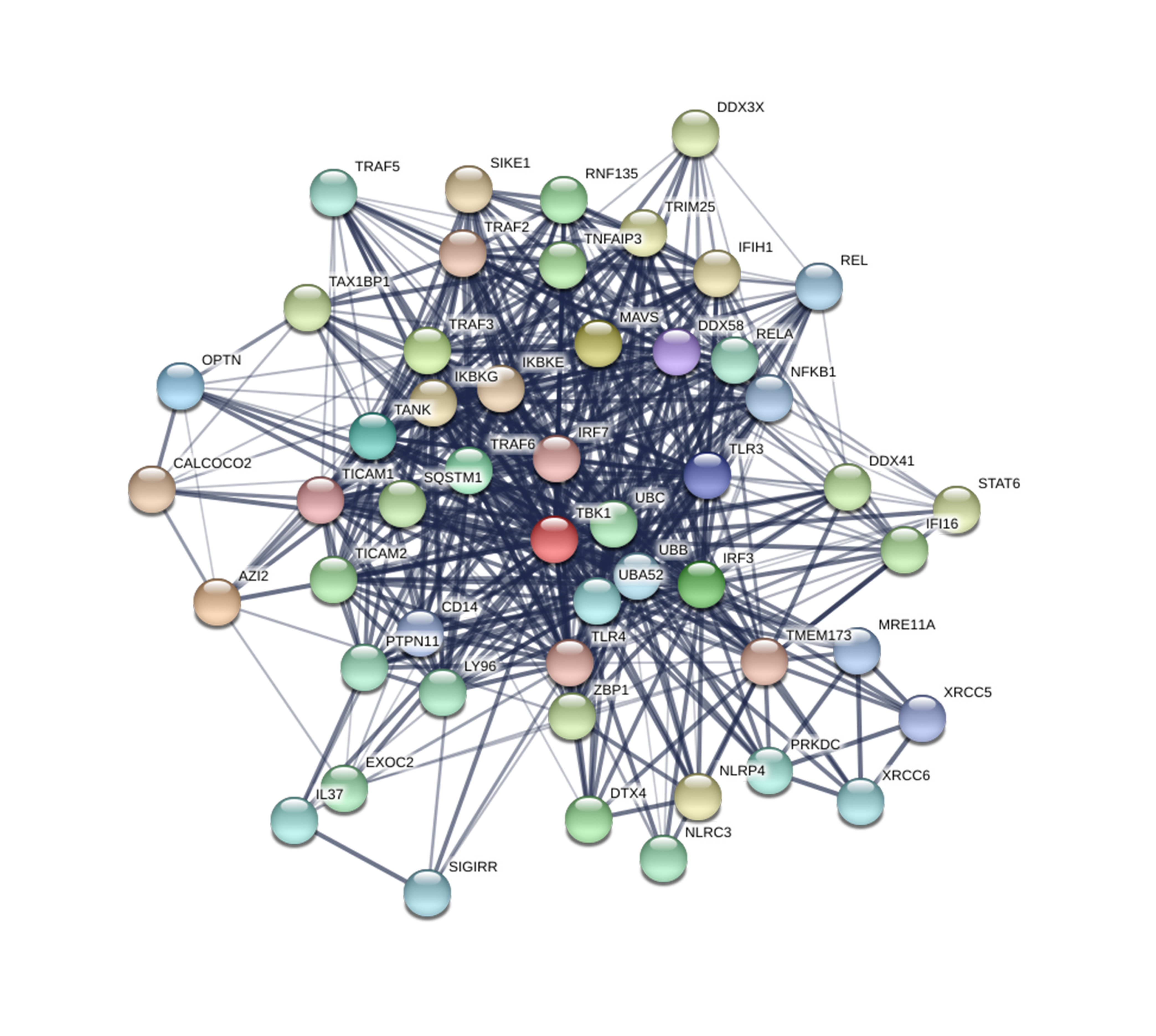

Supplement: Supplementary Figure 4 — Co-expressed genes with TBK1 among patients with HCC. [file Image_4.tif]

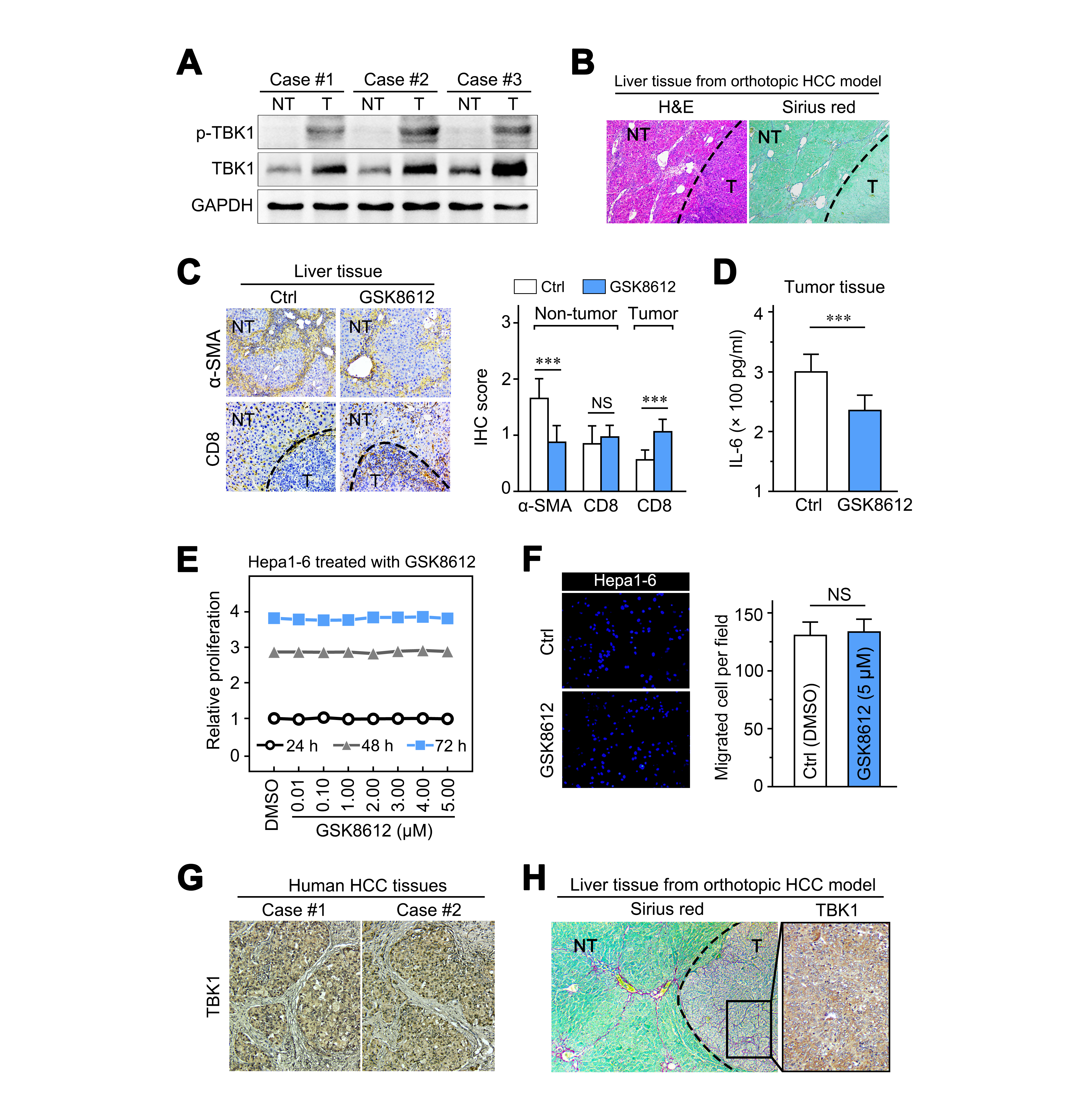

Supplement: Supplementary Figure 5 — TBK1 antagonist improved the immune infiltrates in HCC and attenuated liver fibrosis and tumor inflammation. (A) The expression of TBK1 and p-TBK1 in human HCC tissues and non-tumor liver tissues. (B) The pathological features of liver tissue from HCC mouse model. (C) Representative images of IHC staining with α-SMA and CD8 in liver tissues from control and treatment group (Left panel); statistical analysis of their IHC score (Right panel). (D) The level of IL-6 in HCC tissues were examined by ELISA. (E) The CCK8 and (F) Transwell assays used to measure the effect of GSK8612 on Hepa1-6 proliferation and migration. (G) TBK1 expression in tumor stroma of human HCC tissues. (H) In the liver tissues of C57BL/6 mouse model, TBK1 expression in tumor stroma indicated by Sirius red staining. NT = Non-tumor liver tissue, T = Tumor, NS = not significant; *, P < 0.05; **, P < 0.01; ***, P < 0.001. [file Image_5.tif]
