# Supplementary material for: TANK-Binding Kinase 1 (TBK1) Serves as a Potential Target for Hepatocellular Carcinoma by Enhancing Tumor Immune Infiltration
Source: Front Immunol. 2021 Feb 18;12:612139. doi: 10.3389/fimmu.2021.612139 (PMC7930497; doi:10.3389/fimmu.2021.612139)
Supplement: Supplementary file 6 [file Table_1.docx]

| **Cancer** | **Subtype** | **Fold change** | ***P* value** | **Adjusted**  ***P* Value** | **Reference**  **(PMID)** | **GEO accession**  **number** |
| --- | --- | --- | --- | --- | --- | --- |
| Breast | Ductal Breast Carcinoma in Situ | 1.434 | <0.001 | 0.009 | 19187537 | GSE14548 |
| Brain | Oligodendroglioma | -1.569 | <0.001 | <0.001 | 16616334 | GSE4290 |
| Cervical | Cervical Squamous Cell Carcinoma | 1.428 | <0.001 | <0.001 | 18191186 | GSE7410 |
|  | Cervical cancer | 4.287 | <0.001 | <0.001 | 17510386 | GSE6791 |
| Colorectal | Rectal carcinoma | 1.504 | <0.001 | <0.001 | 18171984 | GSE8671 |
| Gastric | Gastric Mixed Adenocarcinoma | 1.727 | <0.001 | <0.001 | 19081245 | GSE13911 |
| Head and neck | Nasopharyngeal Carcinoma | 1.651 | <0.001 | <0.001 | 17119049 | GSE12452 |
| Kidney | Clear Cell Renal Cell Carcinoma | 1.784 | <0.001 | <0.001 | 17699851 | GSE6344 |
|  | Renal Pelvis Urothelial Carcinoma | 1.649 | <0.001 | <0.001 | 16115910 | GSE15641 |
| Leukemia | T-Cell Prolymphocytic Leukemia | 2.543 | <0.001 | 0.012 | 17713554 | GSE5788 |
| Liver | Hepatocellular Carcinoma | 1.512 | 0.006 | 0.037 | 22689435 | GSE50579 |
| Pancreatic | Pancreatic Ductal Adenocarcinoma | 1.656 | <0.001 | <0.001 | 19260470 | GSE15471 |
