# Supplementary material for: TANK-Binding Kinase 1 (TBK1) Serves as a Potential Target for Hepatocellular Carcinoma by Enhancing Tumor Immune Infiltration
Source: Front Immunol. 2021 Feb 18;12:612139. doi: 10.3389/fimmu.2021.612139 (PMC7930497; doi:10.3389/fimmu.2021.612139)
Supplement: Supplementary file 7 [file Table_2.docx]

|  | OS (n=169) | | | | |
| --- | --- | --- | --- | --- | --- |
|  | Univariate analysis | |  | Multivariate analysis | |
| Characteristics | Hazard | *P* value | | 1Hazard | *P* value |
| **Age (year)** |  |  |  | |  |
| ≥60 *vs.* <60 | 1.696 (0.926--3.106) | 0.087 |  | |  |
| **Gender** |  |  |  | |  |
| Male *vs.* Female | 0.606 (0.335--1.098) | 0.099 |  | |  |
| **Platelet to albumin ratio** |  |  |  | |  |
| High *vs.* Low | 1.264 (0.688--2.322) | 0.450 |  | |  |
| **Liver fibrosis** |  |  |  | |  |
| Cirrhosis vs Non-Cirrhosis | 1.117 (0.603--2.031) | 0.744 |  | |  |
| **AFP** |  |  |  | |  |
| ≥400 *vs.* <400 | 1.319 (0.677--2.571) | 0.415 |  | |  |
| **Vascular invasion** |  |  |  | |  |
| Yes *vs.* No | 1.982 (1.074--3.658) | **0.029** | 1.544 (0.818--2.917) | | 0.180 |
| **Tumor grade** |  |  |  | |  |
| 3+4 *vs.* 1+2 | 1.584 (0.882--2.846) | 0.123 |  | |  |
| **Tumor stage** |  |  |  | |  |
| III+IV *vs.* I+II | 2.066 (1.110--3.846) | **0.022** | 1.923 (1.027--3.601) | | **0.041** |
| **TBK1 expression** |  |  |  | |  |
| High *vs.* Low | 2.784 (1.438--5.395) | **0.002** | 2.473 (1.253--4.881) | | **0.009** |
